# Plate fixation combined with suture bridge technique for proximal humeral fracture-dislocation with displaced greater tuberosity: a case series

**DOI:** 10.1097/RC9.0000000000000192

**Published:** 2026-02-05

**Authors:** Ryogo Furuhata, Atsushi Tanji, Satoshi Oki, Noboru Matsumura

**Affiliations:** a Department of Orthopaedic Surgery, Ashikaga Red Cross Hospital, Ashikaga-shi, Tochigi, Japan; b Department of Orthopaedic Surgery, Keio University School of Medicine, Shinjuku-ku, Tokyo, Japan

**Keywords:** anchor fixation, case series, locking plate, outcome, proximal humeral fracture

## Abstract

**Introduction:**

Plate fixation is an established procedure for proximal humeral fractures involving the greater tuberosity; however, concomitant glenohumeral dislocation increases the risk of secondary displacement of the greater tuberosity fragments. The suture bridge technique has produced satisfactory outcomes for greater tuberosity fractures; however, little is known about the outcomes of combining plate fixation with the suture bridge technique. We report the surgical technique and outcomes of plate fixation combined with the suture bridge technique for proximal humeral fracture-dislocations with displaced greater tuberosity.

Highlights

- We report a plate fixation combined with suture bridge technique for proximal humeral fracture-dislocations.
- Additional suture bridge fixation enhances the stablility of greater tuberosity fragment.
- The technique can be applicable to cases with a high risk of postoperative greater tuberosity displacement.

**Presentation of case:**

We retrospectively identified six patients who underwent plate fixation combined with a suture bridge technique. Using the deltopectoral approach, we reduced the fractured fragments. We inserted medial anchors into the proximal margin of the humeral fracture surface and distal anchors into the shaft, and fixed the greater tuberosity fragments using the suture bridge technique. The fractured fragments were fixed using a locking plate. We evaluated postoperative shoulder functional scores and complications. The mean Constant score was 86, and the American Shoulder and Elbow Surgeons score was 85 at 1 year postoperatively. No major postoperative complications were observed.

**Discussion:**

The procedure yielded satisfactory functional and radiological outcomes. Plate fixation combined with the suture bridge technique allows for more secure fixation of greater tuberosity fragments than conventional procedures, potentially preventing greater tuberosity displacement postoperatively.

**Conclusion:**

This case series presents a new fixation procedure for proximal humeral fractures, which can be advantageous in patients with a high risk of postoperative greater tuberosity displacement.

## Introduction

Plate fixation is a widely used surgical procedure that yields satisfactory outcomes for Neer’s three-part proximal humeral fractures involving the greater tuberosity^[^1–3^]^; however, secondary displacement of the greater tuberosity fragment can occur postoperatively^[^4,5^]^. In particular, a greater tuberosity fracture complicated by glenohumeral dislocation is a risk factor for postoperative greater tuberosity displacement[6]. Residual greater tuberosity displacement causes poor postoperative shoulder functional outcomes^[^4,7^]^; therefore, preventing postoperative displacement of the greater tuberosity is crucial in proximal humeral fracture-dislocations.

Recent studies have reported that the suture bridge technique using suture anchors produces satisfactory functional and radiological outcomes in isolated greater tuberosity fractures^[^8–15^]^. These outcomes are comparable with those of plate fixation[14], and the suture bridge technique can address comminuted greater tuberosity fractures that are unsuitable for plate fixation^[^8–14^]^. These reports raise the possibility that combining plate fixation with the suture bridge technique may provide a more stable fixation for various types of greater tuberosity fragments. However, little is known about the outcomes of combining plate fixation with the suture bridge technique[16].

In this case series, we report the surgical technique and outcomes of plate fixation combined with the suture bridge techniques in six cases of proximal humeral fracture-dislocation with a displaced greater tuberosity. This case series is reported in accordance with PROCESS 2025 guideline[17].

## Case series presentation

### Patient selection

This retrospective case series included patients who underwent plate fixation combined with the suture bridge technique for proximal humeral fractures at two general hospitals between 2019 and 2024. The procedure was performed in cases that met the following criteria: glenohumeral dislocation or after reduction of glenohumeral dislocation, greater tuberosity displacement ≥5 cm, and minimal head-shaft displacement (<1 cm). We included patients who were followed up for 1 year postoperatively. We excluded patients with isolated greater tuberosity fractures, paralysis of the affected upper limb, and previous surgery on the affected shoulder.

In this study, we identified six patients (three women and three men) who met the inclusion and exclusion criteria (Table 1).

**Table 1:** Details of patients who underwent plate fixation combined with suture bridge technique for proximal humeral fracture-dislocation with displaced greater tuberosity.

Age/Sex	Affected side	Fracture type	Operative time (min)	Blood loss (g)	Constant score	ASES score	Complication
70/Male	Right	Avulsion	124	220	84	78	-
63/Male	Right	Split	122	250	100	100	-
90/Female	Right	Avulsion	176	67	70	72	-
72/Male	Right	Split	125	79	84	80	- (Greater tuberosity resoprtion)
82/Female	Right	Split	62	20	98	95	-
80/Female	Right	Split	118	100	82	81	-

### Surgical procedure

**Figure 1.**
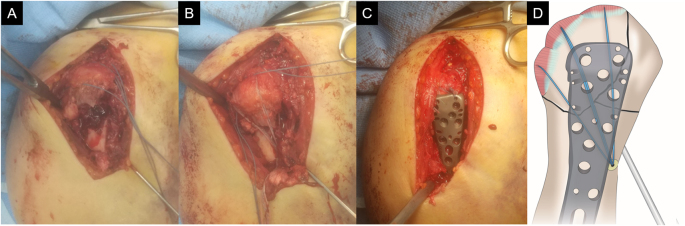
Intraoperative findings of plate fixation with the suture bridge technique. After reduction of the humeral head fragment and humeral shaft, fixation was performed using a Kirschner wire (A). Medial anchors were inserted into the proximal margin of the humeral fracture surface and a distal anchor into the humeral shaft, securing the greater tuberosity fragment with the suture bridge technique (B). The fracture fragments were then fixed using a locking plate (C). An illustration of the procedure is shown (D).

**Figure 2.**
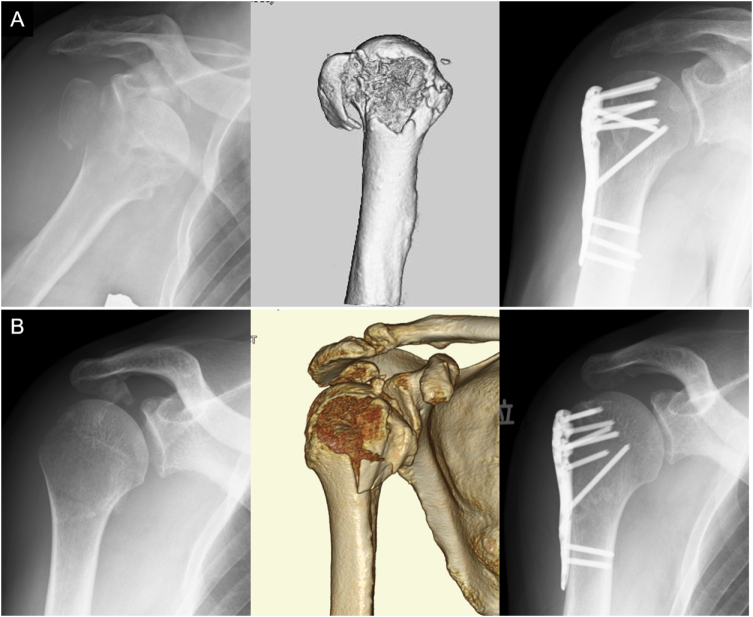
Radiographs taken before and after surgery. (A) 72-year-old man presented with right proximal humeral fracture-dislocation with split fracture of greater tuberosity (left panel). After reduction, computed tomography revealed a proximal humeral fracture with a displaced greater tuberosity fragment and minimal head-shaft displacement (middle panel). Postoperative radiograph of osteosynthesis using a plate and suture bridge technique (right panel). (B) 70-year-old man presented to our hospital after undergoing closed reduction for proximal humeral fracture-dislocation at the other hospital (left panel). Computed tomography showed a proximal fracture-dislocation with an avulsion fracture of greater tuberosity (middle panel). Postoperative radiograph after osteosynthesis using a plate and suture bridge technique (right panel).

All surgeries were performed under general anesthesia by an orthopedic surgeon with over 10 years of experience in shoulder surgery, with the patient in the beach-chair position. The deltopectoral approach was used. In cases in which the dislocation had not been reduced, reduction was performed under fluoroscopy. First, the humeral head fragment and shaft were reduced and temporarily fixed with a 2.0-mm Kirschner wire (Fig. 1A). No. 2 FiberWire (Arthrex, Naples, FL, USA) was passed through the rotator cuff tendons to reduce the greater tuberosity fragment. Depending on the fragment size, two to three JuggerKnot® anchors (Zimmer Biomet, Warsaw, IN, USA) were inserted into the proximal margin of the humeral fracture surface. The suture strands were then passed through the rotator cuff tendons. One to two Quattro® Link Knotless Anchors (Zimmer Biomet, Warsaw, IN, USA) were inserted into the humeral shaft, and the greater tuberosity fragments were fixed using the suture bridge technique (Fig. 1B). Subsequently, the fracture fragments were fixed using the MODE Proximal Humeral Plate® (MDM, Tokyo, Japan) or the ALPS® plate (Zimmer Biomet, Warsaw, IN, USA) (Fig. 1C and 1D). After plate fixation, one FiberWire attached to the supraspinatus tendon and another attached to the infraspinatus tendon were secured through a plate hole. The postoperative radiographs are shown in Fig. 2.

Passive range-of-motion exercises were started on postoperative day 3, and active range-of-motion exercises were started 4 weeks postoperatively. Rotator cuff exercises were permitted at 8 weeks postoperatively.

### Outcome measure

We evaluated the functional and radiological outcomes 1 year postoperatively. Shoulder functional outcomes were assessed using the Constant score[18] and the American Shoulder and Elbow Surgeons (ASES) score[19]. The postoperative radiological outcomes were evaluated by a single evaluator using radiographs to assess complications (screw penetration, avascular necrosis, varus progression, and greater tuberosity reduction loss). Reduction loss was defined as varus progression >10° in the head-shaft angle[20]. Greater tuberosity reduction loss was defined as the summit of the greater tuberosity being <5 mm below the summit of the humeral head on the anteroposterior radiograph in neutral rotation[4].

For the six patients we operated in this study, the mean operative time was 121 min, and the mean blood loss was 123 g. The mean Constant score was 86, and the mean ASES score was 85 at 1 year postoperatively. No postoperative complications were observed; however, resorption of the greater tuberosity occurred in one patient (Table 1).

## Discussion

In this case series, plate fixation combined with the suture bridge technique provided satisfactory functional and radiological outcomes for proximal humeral fracture-dislocations with severely displaced greater tuberosity fragments.

The functional outcomes in our series were comparable to those reported in previous studies of plate fixation for proximal humeral fracture-dislocations^[^21–23^]^. Furthermore, although plate fixation for three-part fractures involving the greater tuberosity has been associated with postoperative malposition with a frequency of 25–26%^[^4,5^]^, no such malposition was observed in our case series.

Traditionally, threading sutures are passed through the rotator cuff and tied to the suture holes of a plate, which may prevent postoperative secondary displacement of the greater tuberosity during plate fixation of proximal humeral fractures. However, these rotator cuff sutures generate tractional stress along the direction of the rotator cuff muscles and cannot compress the greater tuberosity fragments onto the footprint area[24]. A previous biomechanical study demonstrated that additional rotator cuff sutures neither enhanced the stability of the greater tuberosity fragment nor prevented displacement[25]. More recently, plate fixation combined with suture anchor fixation has demonstrated superior biomechanical properties compared with plate fixation using rotator cuff sutures[24]. Several clinical studies have reported that plate fixation combined with suture anchor fixation yields better functional outcomes and lower complication rates than plate fixation alone for isolated greater tuberosity or three-part proximal humeral fractures^[^26–28^]^. This anchor suture technique provides a force perpendicular to the direction of the displacement force, thereby compressing the greater tuberosity fragments onto the footprint area, in contrast to the rotator suture technique[24]. However, these studies^[^24,26–28^]^ reported procedures in which sutures were passed from a medial suture anchor thread to the suture holes of the plate. To our knowledge, no previous studies have reported combining plate fixation with a suture bridge technique, as performed in our series.

Our surgical procedure offers three advantages over conventional techniques. First, the additional suture bridge technique allows compression of the entire greater tuberosity fragment onto the humeral fracture surface^[^8–10^]^, potentially providing superior stability compared with previous procedures combining plates and suture anchors. Specifically, sutures inserted into the distal anchors can function as a tension band to effectively buttress the tuberosity fragments against the humeral fracture surface[8]. Second, achieving intraoperative stability of the greater tuberosity fragments with the suture bridge technique can facilitate plate placement and screw insertion. Conventional plate fixation for three-part fractures involving the greater tuberosity is technically challenging because plate placement and screw insertion must be performed while simultaneously reducing the fragments by traction of the rotator cuff sutures. Third, additional suture bridge fixation may prevent postoperative subacromial impingement by allowing placement of the plate in a more distal position[26].

However, this procedure has two limitations. First, insertion of certain proximal screws may risk entanglement with sutures secured by the suture bridge technique. Second, additional suture anchor fixation carries the risk of complications such as anchor pullout or iatrogenic fracture during anchor insertion.

Our procedure may be applicable to proximal humeral fractures with a high risk of postoperative secondary displacement of the greater tuberosity. For cases with a high risk of secondary displacement of isolated greater tuberosity fractures – such as comminuted fractures, poor bone mineral density, or concomitant glenohumeral dislocations[6] – our procedure may help prevent fixation failure of the fragments. However, further studies are needed to verify this hypothesis.

This case series has some limitations. First, the small sample size and retrospective design may have introduced selection bias, limiting the generalizability of the findings. Second, as this was a nonrandomized retrospective study without a control group, we could not compare outcomes with and without the suture bridge techniques. Third, we could not assess long-term outcomes.

## Conclusion

This case series provides new insights into surgery for proximal humeral fracture-dislocation with a displaced greater tuberosity. Incorporating a suture bridge technique with plate fixation may provide more secure fixation of greater tuberosity fragments than conventional methods, potentially preventing postoperative displacement.

## Data Availability

Data supporting the findings of this study are available from the corresponding author upon request.

## Guarntor

Royogo Furuhata.
